# The Relationship between Transformational Leadership and Staff Nurse Retention in Hospital Settings: A Systematic Review

**DOI:** 10.1155/2023/9577200

**Published:** 2023-11-25

**Authors:** Nathalie Conroy, Declan Patton, Zena Moore, Tom O'Connor, Linda Nugent, Rosemarie Derwin

**Affiliations:** ^1^School of Nursing and Midwifery, Royal College of Surgeons in Ireland (RCSI) University of Medicine and Health Sciences, Dublin, Ireland; ^2^Beaumont Hospital, Beaumont, Dublin 9, Ireland; ^3^Skin Wounds and Trauma (SWaT) Research Centre, RCSI University of Medicine and Health Sciences, Dublin, Ireland; ^4^School of Nursing and Midwifery, Griffith University, Brisbane, Queensland, Australia; ^5^Fakeeh College of Health Sciences, Jeddah, Saudi Arabia; ^6^Faculty of Science, Medicine and Health, University of Wollongong, Wollongong, Australia; ^7^School of Health Sciences, Faculty of Life and Health Sciences Ulster University, Ireland; ^8^Department of Public Health, Faculty of Medicine and Health Sciences, Ghent University, Ghent, Belgium; ^9^Lida Institute, Shanghai, China; ^10^Cardiff University, Cardiff, Ireland

## Abstract

**Aim:**

This systematic review aimed to synthesise the relationship between transformational leadership style and staff nurse retention in hospital settings.

**Background:**

It is known that globally there is a shortage of nurses. Thus, nurse retention and organisational commitment have never been more critical. Nurse managers are responsible for staff retention. Therefore, nurse managers could reduce staff turnover by adopting the “right leadership style.”

**Methods:**

Systematic review, following the guidance of PRISMA. Databases CINAHL, MEDLINE, PubMed, PsychInfo, Cochrane Central Register, and Embase were searched between 27^th^ December 2021 and 22^nd^ June 2023 to find relevant publications. Relevant studies were hand searched in January 2022 and June 2023 to source further potential evidence. A total of twelve articles were retrieved.

**Results:**

Twelve studies were included in this review, including six cross-sectional studies, two correlational studies, two cross-sectional correlational studies, and two surveys. In relation to retention, the primary outcome, data from eleven of the twelve studies reported statistical significance favouring transformational leadership improving staff retention. One study reported a statistically nonsignificant improvement in retention.

**Conclusion:**

There is evidence to suggest that transformational leadership may have a positive and significant relationship with staff nurse retention, job satisfaction, and quality of care. *Implications for Nursing Management*. Nurse managers should attend leadership and management training programs. This will allow them to understand and practice transformational leadership which may have a positive connection with staff nurse retention.

## 1. Introduction and Background

The World Health Organisation [1] has reported that the shortage of healthcare workers is a global concern, particularly nurses and midwives, representing more than 50% of the current shortage of health workers. Staff turnover rates differ significantly in high-income countries, with the highest rate of 44.3% reported in New Zealand, 26.8% in the USA, 23% in Israel, 19.9% in Canada, and 15.1% in Australia [2]. Moreover, in Ireland, the HSE [3] highlighted that the staff nurse turnover rate was 7.7% in 2021, above the national average of 6.4% [4], highlighting the increased struggle for staff retention. The explanation for this is unclear, thus, warranting the need for further research to determine the reason for this. It is estimated that the global nursing shortage will reach 1.05 million by the end of 2022, keeping with a nurse turnover rate of 18.69% [5]. Numerous studies imply that 4% to 54% of nurses globally intend to quit nursing [6], highlighting concern over inadequate staff and adverse patient outcomes [7]. A growing body of evidence suggests that nurse manager leadership influences nurse retention [8, 9].

Employees are vital commodities of an organisation; thus, this becomes the main area of focus where leadership should lead in ways that improve staff retention [10]. In recent years, leadership has become an important concept in nursing; therefore, leadership styles are required to reduce waste, cost, confusion, and error [11]. It is well documented that leadership styles used by nurse managers play an essential role in nurses' commitment to their workplace [12]. In addition, although job satisfaction can be increased by extrinsic means such as a pay rise, nurse managers can improve job satisfaction by adopting the right leadership style [13]. It is suggested that almost one-third of the level of job satisfaction of nursing staff can be increased by managers just manipulating their leadership behaviours [13]. Thus, nurse managers could cost-effectively retain their staff by displaying the “right leadership style” [13]. Newstrom [14] describes leadership styles as how individuals provide direction, implement plans, and motivate staff. It is proposed that managers who adopt the transformational leadership (TL) style transform their followers' ideas about what is important, inspiring them to see opportunities and challenges in a positive light [15].

Transformational leadership has been compartmentalised into four main components: idealised influence, inspirational motivation, intellectual stimulation, and individualised consideration [16]. Idealised influence occurs when leaders bring about trust and respect by acting as role models to their followers [17]. Second, inspirational motivation occurs when nurse managers enable their staff to achieve the organisation's mission and personal goals [18]. Third, intellectual stimulation occurs when nurse managers encourage their staff to develop new ideas and keep learning through courses or evidence-based materials [18]. Finally, individualised consideration occurs when nurse managers encourage individual staff members by helping them and supporting and providing positive feedback [18]. Therefore, a transformational leader is a leader who can promote the interest of staff and facilitate the commitment of staff to the mission of the organisation [19]. It is clear that the leadership practices of nurse managers can positively or negatively affect outcomes for organisations, staff, and patients [20].

Increased staff turnover in a hospital may result in increased overtime, fatigue, stress, and poor job satisfaction among remaining nurses [21]. Furthermore, it upsets continuity of care, resulting in reduced quality of care and safety, potentially increasing the risk of medication errors, falls, and healthcare-associated infections [21]. Thus, increased staff turnover is of great concern for nurse managers. Moreover, increased staff turnover may also negatively affect hospital budgets [22]. Using the original Nursing Turnover Cost Calculation Methodology, yearly costs associated with nurse turnover were approximately $48,790 in Australia, $20,561 in the US, $26,652 in Canada, and $23,711 in New Zealand [23, 24]. These costs highlight the importance of nurse retention; hence, it is vital to explore the relationship of TL style on staff nurse retention in hospital settings. Effective leadership is considered an essential part of staff nurse retention [25]. Thus, conducting this systematic review is vital to synthesise the link between TL and staff nurse retention in hospital settings to reduce staff turnover. Therefore, this systematic review aims to synthesise the body of knowledge on the relationship between TL and staff nurse retention in hospital settings.

## 2. Methods

**Figure 1:**
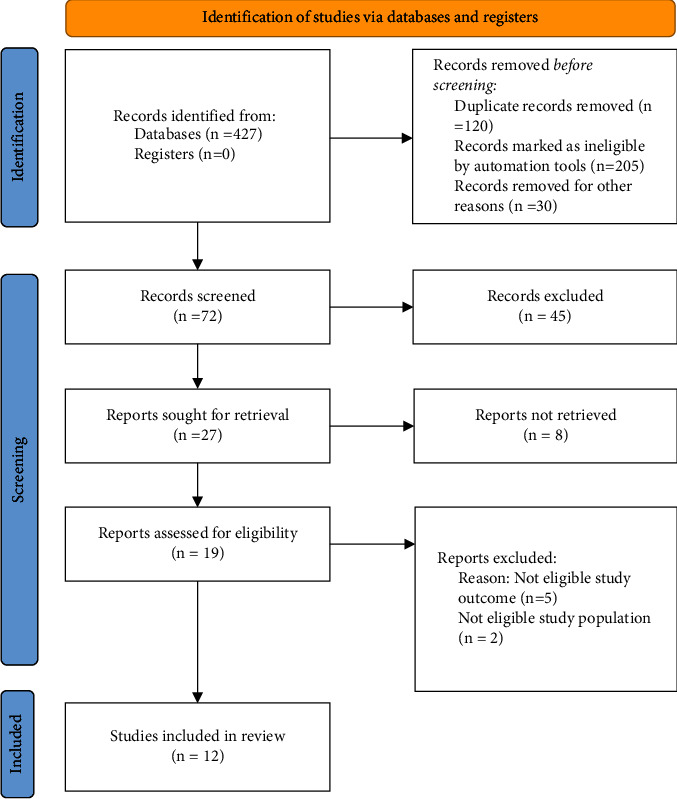
PRISMA flow diagram.

A systematic review was undertaken using the Preferred Reporting Items for Systematic Reviews and Meta-Analysis (PRISMA) checklist (Figure 1). The review question was formulated using the PICO mnemonic. The population being considered was staff nurses working in hospital settings, the intervention was transformational leadership which was compared with other leadership styles. The primary outcome was staff retention, and the secondary outcomes were job satisfaction and quality of care. Thus, the research question was “What is the relationship between transformational leadership style and staff nurse retention in hospital settings? This question was formulated as it was an area of interest to the authors.”

Table 1 details the inclusion and exclusion criteria.

### 2.1. Search Strategy

A systematic search was conducted to ensure all available evidence to answer the question was included. The searches were conducted from December 2021 to June 2023. The following databases were searched: CINAHL, MEDLINE, PubMed, PsychInfo, Cochrane Central Register, and Embase. Keywords and phrases included were as follows: transformational AND leadership OR management AND style OR method OR approach AND nurse OR caregiver OR healthcare professional OR healthcare worker AND retention OR turnover OR commitment OR intent to stay OR organisational commitment OR affective commitment OR reduced predicted turnover OR turnover intention OR anticipated turnover OR intention to leave AND hospital OR acute care setting OR acute care facility. The English language limitation was applied. The reference list of identified studies was hand-searched for suitable studies and citations.

### 2.2. Data Extraction and Data Analysis

One review author independently extracted data from eligible studies using a data extraction sheet and table; this was validated by five authors (see Table 2 for data extraction). The data extraction table included authors, year, country, study setting, study design, population and sample size, results, primary outcome, and secondary outcomes (Table 2). Meta-analysis was not feasible due to the heterogeneity of the studies included. Therefore, a narrative description of the studies was undertaken.

### 2.3. Quality Appraisal

The quality of the selected studies was appraised by six independent reviewers, who assessed the internal and external validity and determined the bias affecting the methodological quality. Furthermore, the Critical Appraisal Checklist (EBL) devised by Glynn [34] was also used to appraise the included studies. Accordingly, the studies were appraised under the following headings: population, data collection, study design, and results. Applying this tool, the study quality in each category is invalid with a final score of <75. Therefore, the studies that produced results of “Yes” ≥75% or, “No + Unclear” ≤25% were considered good quality. The score from each section was calculated at the end to indicate the study's validity.

## 3. Results

The initial search yielded 427 articles. The six authors worked in pairs for the filtration process and after removing duplicates, 307 remained. Upon removal of ineligible studies, 72 were screened, of which 45 were excluded. Of the remaining 27, 8 studies were not retrieved. Following this, 19 full-text articles were rigorously screened for eligibility. 7 were excluded for valid reasons. 5 articles had a noneligible study outcome, and 2 had a noneligible study population. This resulted in 12 studies meeting the inclusion criteria for this systematic review (Figure 1).

### 3.1. Description of Included Studies

Twelve studies met the inclusion criteria for this systematic review. The research was conducted in hospital settings across many different countries, namely, Saudi Arabia [12, 30], Canada [26], Ghana [2, 13], Japan [27, 33], Jordan [28], China [29], Iran [31], Philippines [32], and Turkey [10]. The mean sample size in the twelve studies was 535 participants, ranging from 219 to 1,617 participants. Participants in one of the twelve studies were a mix of registered nurses and nurse managers. The study's characteristics are presented in the data extraction table (Table 2).

#### 3.1.1. Data Collection Instruments

Table 3 represents the details of various data collection instruments used in the included studies. All the data collection methods were deemed reliable and valid as Cronbach's alpha scores were all greater than 0.50 [35]. Two of the twelve studies did not detail Cronbach's alpha score; however, both studies reported that their instruments were reliable.

#### 3.1.2. Primary Outcome

The primary outcome, staff retention, was measured in all twelve studies. Overall, eleven of the twelve studies demonstrated that there was a positive relationship between TL style and staff nurse retention in hospital settings. The results are illustrated in Table 4. Lavoie-Tremblay et al. [26] revealed that TL style negatively and significantly predicted the intention to quit (*r* = −0.39, *P* ≤ 0.05). Asamani et al. [13] found that there was a weak but significant positive correlation between TL style and staff nurses' intention to stay (*r* = 0.221, *P* ≤ 0.001). Kodama et al. [27] reported that TL style was significantly and positively related to affective commitment (OR = 2.23, 95% CI: [1.31–3.80]). Abualrub and Nasrallah [28] discovered that increased staff retention was associated with TL style (*r* = 0.391, *P* ≤ 0.001). Wang et al. [29] reported that TL style was positively correlated with nurse retention (*P* ≤ 0.001, 95% CI: [0.269–0.478]). Al-Yami et al. [30] revealed that TL style and organisational commitment were positively related (*r* = 0.364, *P* ≤ 0.01). Pishgooie et al. [31] found that there was a positive correlation between TL style and anticipated turnover (*r* = −0.22, *P* ≤ 0.001). Labrague et al. [32] discovered that TL style correlated significantly with organisational turnover intention (*r* = −0.08, *P* ≤ 0.05). Magbity et al. [2] reported a significant correlation between TL style and nurses' turnover intention (*r* = −0.377). Suliman et al. [33] found that TL style had a significant effect on nurse turnover (*P* ≤ 0.001). Yücel [10] discovered that TL style significantly negatively predicted turnover intention (*P* ≤ 0.001). These results indicate that TL style has a statistically significant positive connection with staff nurse retention in hospital settings. Abualrub and Alghamdi [12] was the only study to report that the relationship between TL style and staff retention was statistically insignificant (*P* ≤ 0.14), indicating that there was no relationship between TL style and staff nurses' retention at work.

#### 3.1.3. Secondary Outcomes

Three studies found a positive connection between TL style and job satisfaction. The results are illustrated in Table 5, while one study found that TL style resulted in high quality of care.

A multiple linear regression analysis was used to determine if the demographic characteristics (model 1) and the nurse managers' leadership styles (model 2) significantly accounted for the levels of job satisfaction. The results in the three articles revealed that TL style was linked to nurses' job satisfaction. Abualrub and Alghamdi [12] reported a positive significant moderate correlation between TL style and nurses' job satisfaction (*r* = 0.45, *P* ≤ 0.001). Asamani et al. [13] found that TL style of nurse managers was positively correlated with staff nurses' levels of job satisfaction (*r* = 0.462, *P* ≤ 0.001). Labrague et al. [32] also discovered that TL style correlated significantly with job satisfaction (*r* = 0.37, *P* ≤ 0.001). These results suggest that TL style has a statistically significant positive link to staff nurses' job satisfaction.

Lavoie-Tremblay et al. [26] investigated the link between nurse manager leadership styles and quality of care. Quality of care was measured on a 4-item scale [36]. This scale was deemed reliable as it had a Cronbach's alpha score of 0.84. Lavoie-Tremblay et al. [26] discovered that TL style had a positive and significant connection with quality of care (*P* ≤ 0.001).

### 3.2. Quality Appraisal

The results from the quality appraisal are presented in Table 6. All studies were deemed valid, except for Magbity et al. [2] who showed issues with the choice of population and the results section. In [2], the inclusion and exclusion criteria were not clearly outlined, and it was unclear whether informed consent was obtained from the participants. It was also unclear whether there was external validity. Suggestions for further research were not provided, and subset analysis was a major focus.

## 4. Discussion

The primary aim of this systematic review was to examine the relationship between TL style and staff nurse retention in hospital settings. The secondary outcomes were presented as job satisfaction and quality of care. The results of eleven of the twelve studies indicate that TL style positively affects staff nurse retention [2, 10, 13, 26–33]. Moreover, three of the twelve studies suggest that TL style has a positive and significant link with job satisfaction [12, 13, 32]. One study discovered that TL style also has a positive and significant connection to quality of care [26].

The study conducted by Abualrub and Alghamdi [12] indicated that the relationship between TL style and staff nurse retention was statistically insignificant. The results from this study may suggest that TL style has a minimal link to nurse retention. However, this study was the most outdated, which may explain why this was the only study with this outcome. Upon review, Asamani et al. [13] implied that there was a weak but significant connection between TL style and staff nurse retention (*r* = 0.221, *P* < 0.001) as this study found a stronger correlation between participative leadership and staff nurse retention (*r* = 0.243, *P* < 0.001).

Overall, the results from this systematic review indicate that TL style has a positive relationship with staff nurse retention. In support of this, multiple past studies have suggested that nurses are seeking challenges in their work [37]; they want to be encouraged, respected, and recognised (Lavoie-Tremblay et al., 2010) and require feedback on their performance [38, 39]. Moreover, Hutchinson et al. [40] implied that a supportive work environment might be vital in retaining nurses. Likewise, a high level of collaboration in nursing units was linked with retention [41]. These elements, which concern the needs of nurses, can be facilitated through TL practices [26] and thus result in staff retention. When considering mixed results such as this, it would be appropriate to question the specific elements of TL style that may or may not significantly link to staff retention. Furthermore, it may be relevant to compare different leadership styles and their relationship with staff retention to determine which style is best related to staff nurse retention; thus, this requires further research.

Concerning the secondary outcome of this systematic review, the three studies that measured job satisfaction [12, 13, 32] highlighted that TL style has a significant positive connection with job satisfaction. Both Abualrub and Alghamdi [12] and Asamani et al. [13] reported that levels of job satisfaction among nurses were generally moderate. Thus, it may be implied that nurses across different countries are generally not satisfied with their jobs, a situation that can potentially reduce productivity and worsen the current shortage of nurses [13]. However, as previously mentioned, TL had a positive correlation with job satisfaction. This suggests that the adoption of TL style by nurse managers may result in an increase in the level of nursing staff and job satisfaction [12, 13, 32].

Besides the results of the present systematic review, Cummings et al. [42] also conducted a systematic review and found that TL style had a strong connection with job satisfaction, productivity, and retention. Thus, participatory management practices and a nurse-friendly work environment, which can be accomplished with a nurse manager who adopts a TL style, can be related to staff retention and job satisfaction [21, 43]. The potential positive significance of nurse retention, considering that improved job satisfaction could reduce turnover, must be recognised as another area for further research.

Interestingly, Lavoie-Tremblay et al. [26] reported that TL style has a significant positive link to quality of care. This was the only study that measured quality of care and found that TL style resulted in high quality of care, another secondary outcome. Excluding the results from this systematic review, previous systematic reviews discovered that TL style was associated with increased patient satisfaction, reduced adverse events, lower patient mortality, and fewer hospital-acquired infections [44, 45].

### 4.1. Limitations

This systematic review was limited by the research available for inclusion. Most of the included studies were cross-sectional and correlational by design, which contributed to the weakness of being unable to determine casual relationships compared with a cohort study [46]. In addition, two studies used the survey method for collecting data which has its own set of disadvantages. It is suggested that one typical issue with employing surveys to gather data is that of missing data [47]. Although there are statistical techniques available to handle missing data, these techniques do not always result in complete accuracy [10]. Furthermore, eight out of the twelve studies utilised convenience sampling [2, 12, 13, 27–29, 32, 33], which is nonrandom and may limit the generalisability of the results. Nonetheless, eleven of the twelve included studies scored highly (>75%) on quality appraisal. Further research should utilise an RCT design to assess the leadership practices of nurse managers (Lavoie-Tremblay et al., 2015). Another limitation of this review is that eleven of the twelve studies included were all pre-COVID-19 [2, 12, 13, 26–33]. Although this was a limitation, both pre-COVID-19 studies and the post-COVID-19 study had similar findings. Furthermore, another limitation to this SR may be that the literature on the original aim was expanded based on the findings; thus, they may be considered an extrapolation from the original aim. Finally, included studies were limited to English, as there was no funding for translation services.

## 5. Conclusion

This review aimed to examine the relationship between TL style and staff nurse retention in hospital settings. The results of this study highlighted the importance of TL style in enhancing staff nurse retention, job satisfaction, and quality of care. Agreeable with the literature, this SR provides support to previous studies connecting TL style to encouraging results in nurses, especially staff nurse retention. Nonetheless, the results are not entirely definitive, as there is a scarcity of primary research related to this issue, highlighting the need for further research in this vital area.

## 6. Implications for Nursing Management

This review has several implications for nursing management. Nurse directors should promote the TL leadership behaviours of nurse managers through leadership training programmes to enhance staff retention. Educational leadership programmes can positively and significantly impact nurse managers' leadership and professional behaviours [48]. Furthermore, in partnership with nurse educators, the regulatory bodies of the nursing profession should develop competencies for nurse managers based on TL and include these competencies in nursing education programmes [28]. The recruitment policies of nurses for leadership roles should be based on these competencies [28].

In addition, it is suggested that magnet hospitals have improved staff retention [49]; this may be because TL style is one component of the magnet model [50]. Thus, magnet hospitals utilise the TL style and have greater staff nurse retention than nonmagnet hospitals [49]. This is important when considering the results from this review as leadership programmes would better educate nurse managers concerning TL style, which can be linked with staff nurse retention, job satisfaction, and quality of care.

## Figures and Tables

**Table 1:** Inclusion and exclusion criteria.

Inclusion criteria	Exclusion criteria
Quantitative research	Qualitative and literature review
Participants who were registered nurses	Nurses who were not registered
Transformational leadership	Studies not including transformational leadership
Measuring retention, turnover, commitment, intent to stay, organisational commitment, affective commitment, reduced predicted turnover, turnover intention, and anticipated turnover	Studies not including retention, turnover, commitment, intent to stay, organisational commitment, affective commitment, reduced predicted turnover, turnover intention, and anticipated turnover
Working in hospital settings	Settings outside the hospital setting
English language	Non-English language
Post-year 2011	Pre-year 2011

**Table 2:** Data extraction.

Authors and year	Country	Study setting	Study design	Population and sample size	Primary outcome	Secondary outcome
Abualrub and Alghamdi [12]	Saudi Arabia	Western region of Saudi Arabia: 6 public hospitals	Correlational design	308 registered nurses. 56% female, 44% male	TL was not linked with nurse retention. *P* ≤ 0.14	TL had a positive effect on job satisfaction. *P* ≤ 0.001
Lavoie- Tremblay et al. [26]	Canada	Quebec hospitals	Cross-sectional	541 registered nurses. 88.2% female, 11.8% male	Positive relationship between TL and nurse retention. *P* ≤ 0.05	TL lead to a high quality of care. *P* ≤ 0.0001
Asamani et al. [13]	Ghana	Eastern region of Ghana: 5 hospitals	Cross-sectional	273 registered nurses. 78% female, 21.3% male	Positive relationship between TL and nurse retention. *P* ≤ 0.001	TL was positively correlated to job satisfaction
Kodama et al. [27]	Japan	Kanto: 4 midsized acute care hospitals	Cross-sectional	396 registered nurses. 93.9% female, 6.1% male	Positive relationship between TL and nurse retention. OR = 2.23	
Abualrub and Nasrallah [28]	Jordan	6 hospitals-a mix of public, private and university-affiliated	Correlational design	285 registered nurses. 56.1% female, 43.9% male	Positive relationship between TL and nurse retention. *P* < 0.001	
Wang et al. [29]	China	Shanghai: 4 general hospitals	Cross-sectional	535 registered nurses, 98.3% female, 1.7% male	Positive relationship between TL and nurse retention. *P* ≤ 0.001	
Al-Yami et al. [30]	Saudi Arabia	Riyadh: 2 biggest hospitals	Survey design	219 registered nurses and nurse managers. 55 nurse managers and 164 staff nurses. 89% female, 11% male	Positive relationship between TL and nurse retention. *P* ≤ 0.001	
Pishgooie et al. [31]	Iran	10 government hospitals	Correlational cross-sectional	1,617 registered nurses. 72.2% female, 27.8% male	Positive relationship between TL and nurse retention. *P* ≤ 0.001	
Labrague et al. [32]	Philippines	Central Philippines: 15 hospitals	Cross-sectional	770 registered nurses. 58.6% female, 41.4% male	Positive relationship between TL and nurse retention. *P* ≤ 0.05	TL positive influence on job satisfaction. *P* ≤ 0.001
Magbity et al. [2]	Ghana	5 hospitals	Cross-sectional	250 registered nurses	Positive relationship between TL and nurse retention. *r* = −0.377	
Suliman et al. [33]	Jordan	North Jordan: 3 public sector hospitals, 1 university-affiliated hospital	Cross-sectional, correlational design	250 registered nurses. 59% female, 41% male	Positive relationship between TL and nurse retention. *P* ≤ 0.001	
Yücel [10]	Turkey	2 private hospitals in Ankara and Istanbul	Survey design	478 participants. 58.3% female, 41.7% male	Positive relationship between TL and staff nurse retention. *P* < 0.001	

**Table 3:** Data collection instruments.

Authors	Leadership measurement	Reliability	Retention measurement	Reliability
Abualrub and Alghamdi [12]	Multifactor Leadership Questionnaire (MLQ)	Cronbach's alpha was 0.87	McCain's Intent to Stay Scale	Cronbach's alpha was 0.80
Lavoie-Tremblay et al. [26]	Global Transformational Leadership (GTL) Scale	Cronbach's alpha was 0.94	O'Driscoll and Beehr Scale	Cronbach's alpha was 0.91
Asamani et al. [13]	Path-Goal Leadership Questionnaire	Cronbach's alpha was 0.701	Intention to Stay Scale	Cronbach's alpha was 0.695
Kodama et al. [27]	MLQ	Cronbach's alpha was 0.87	Affective Commitment Scale	Cronbach's alpha was 0.77
Abualrub and Nasrallah [28]	Leadership Practice Inventory	Cronbach's alpha was 0.97	McCain's Intent to Stay Scale	Cronbach's alpha was 0.88
Wang et al. [29]	Transformational Leadership Scale	Cronbach's alpha was 0.90	Intent to Stay Scale	Cronbach's alpha was 0.79
Al-Yami et al. [30]	MLQ	Cronbach's alpha was >0.60	Organisational Commitment Questionnaire	Cronbach's alpha was 0.77
Pishgooie et al. [31]	MLQ	Cronbach's alpha was 0.90	Anticipated Turnover Scale (ATS)	Cronbach's alpha was 0.73
Labrague et al. [32]	GTL scale	Cronbach's alpha was 0.91	O'Driscoll and Beehr Scale	Cronbach's alpha was 0.92
Magbity et al. [2]	MLQ	Not reported	Turnover Intention Scale	Not reported
Suliman et al. [33]	MLQ	Not reported	ATS	Not reported
Yücel [10]	MLQ	Cronbach's alpha was 0.89	Six-item turnover intention scale	Cronbach's alpha was 0.84

**Table 4:** Primary outcome.

Authors	Staff retention	Pearson correlation value	*P* value	OR	95% confidence interval (CI)
Abualrub and Alghamdi [12]	TL was not linked with nurse retention	*R* = 0.08	*P* ≤ 0.14		
Lavoie-Tremblay et al. [26]	TL style reduced intention to quit	*R* = −0.39	*P* ≤ 0.05		
Asamani et al. [13]	Positive relationship between TL and nurse retention	*R* = 0.221	*P* ≤ 0.001		
Kodama et al. [27]	Positive relationship between TL and nurse retention			2.23	(1.31–3.80)
Abualrub and Nasrallah [28]	TL style was associated with higher levels of retention	*R* = 0.391	*P* ≤ 0.001		
Wang et al. [29]	Positive relationship between TL and nurse retention	*R* = 0.375	*P* ≤ 0.001		(0.269–0.478)
Al-Yami et al. [30]	Positive relationship between TL and nurse retention	*R* = 0.374	*P* ≤ 0.001		
Pishgooie et al. [31]	TL style reduced intention to quit	*R* = −0.22	*P* ≤ 0.001		
Labrague et al. [32]	TL style reduced intent to leave	*R* = −0.08	*P* ≤ 00.05		
Magbity et al. [2]	TL style reduced turnover intention	*R* = −0.377			
Suliman et al. [33]	TL style reduced intention to leave		*P* ≤ 0.001		
Yücel [10]	TL style reduced turnover intention		*P* ≤ 0.001		

**Table 5:** Job satisfaction.

Authors	Job satisfaction (JS)	Correlation value	*P* value
Abualrub and Alghamdi [12]	Positive link between TL style and JS	*R* = 0.45	*P* ≤ 0.001
Asamani et al. [13]	Positive link between TL style and JS	*R* = 0.462	*P* ≤ 0.001
Labrague et al. [32]	Positive link between TL style and JS	*R* = 0.37	*P* ≤ 0.001

**Table 6:** Critical appraisal checklist (EBL) results.

Study	Section A: population	Section B: data collection	Section C: study design	Section D: results	Overall validity
Abualrub and Alghamdi [12]	83% valid	67% valid	100% valid	67% valid	78% valid
Lavoie-Tremblay et al. [26]	100% valid	67% valid	100% valid	67% valid	83% valid
Asamani et al. [13]	83% valid	83% valid	100% valid	50% valid	78% valid
Kodama et al. [27]	83% valid	67% valid	100% valid	67% valid	78% valid
Abualrub and Nasrallah [28]	83% valid	83% valid	100% valid	67% valid	83% valid
Wang et al. [29]	83% valid	67% valid	80% valid	83% valid	78% valid
Al-Yami et al. [30]	83% valid	83% valid	80% valid	67% valid	78% valid
Pishgooie et al. [31]	83% valid	83% valid	100% valid	100% valid	91% valid
Labrague et al. [32]	83% valid	83% valid	80% valid	67% valid	78% valid
Magbity et al. [2]	50% valid	83% valid	80% valid	50% valid	65% not valid
Suliman et al. [33]	83% valid	83% valid	80% valid	83% valid	83% valid
Yücel [10]	83% valid	57% valid	80% valid	67% valid	74% valid

## Data Availability

The data that support the findings of this study are available from the corresponding author upon reasonable request.
